# Sialylation Inhibition Impairs Migration and Promotes Adhesion of GBM Cells

**DOI:** 10.3390/ijms262110708

**Published:** 2025-11-03

**Authors:** Deborah Gargano, Mariangela Calvitto, Antonella Niro, Giuseppe Pepe, Noemi Martella, Alessia Tani, Paolo Rosa, Vittorio Maglione, Giovanni Musci, Antimo Cutone, Sabrina Di Bartolomeo, Eleonora Sgambati

**Affiliations:** 1 Department of Biosciences and Territory, University of Molise, 86090 Pesche, Italy; d.gargano@studenti.unimol.it (D.G.); mariangela.calvitto@unimol.it (M.C.); a.niro2@studenti.unimol.it (A.N.); noemi.martella@unimol.it (N.M.); musci@unimol.it (G.M.); antimo.cutone@unimol.it (A.C.); 2 Neuromed IRCCS, Via Dell’Elettronica, 86077 Pozzilli, Italy; giuseppe.pepe@neuromed.it (G.P.); vittorio.maglione@neuromed.it (V.M.); 3 Department of Experimental and Clinical Medicine, Section of Anatomy and Histology, Imaging Platform, University of Florence, 50134 Florence, Italy; alessia.tani@unifi.it; 4 Department of Medical-Surgical Sciences and Biotechnologies, University of Rome “Sapienza”, Polo Pontino, 04100 Latina, Italy; p.rosa@uniroma1.it

**Keywords:** glioblastoma multiforme, sialic acids, sialyltransferases, 3-Fax-peracetyl-Neu5Ac, cell migration, cell adhesion

## Abstract

Aberrant sialylation has been associated with many types of tumors, characterized by aggressiveness and undifferentiated state. However, not exhaustive investigations have been performed on the sialylation status in glioblastoma multiforme (GBM), the most common primary and lethal malignant brain tumor in humans. Hence, in this study we performed a comprehensive characterization of the sialylation status in GBM evaluating specific sialyltransferases and various types of sialic acids (Sias) in different GBM cell lines. First, through in silico analysis we showed that the sialyltransferases *ST6GAL1*, *ST3GAL2* and *ST8SIA4* are significantly up-regulated in GBM tissues and related to lower patient survival. Then, we evaluated the expression levels of these sialyltransferases and their related Sias and observed a high variability among the different GBM cell lines. In addition, using the pan-sialyltransferase inhibitor 3-Fax, we highlighted the role of sialylation in some of the main oncogenic properties of GBM. Indeed, a significant reduction in mobility and migration capacity along with increased adhesiveness of GBM cells was observed upon sialyltransferases inhibition. Our findings showed that aberrant expression of different Sias types is crucial for cell migration and adhesion ability of GBM cells, suggesting that Sias might represent biomarkers for GBM and be useful to design innovative therapeutic strategies.

## 1. Introduction

Gliomas are primary intracranial tumors arising within the central nervous system (CNS) from glial cells in adult age and account for 80% of malignant brain tumors [1,2,3,4]. Among gliomas, glioblastoma multiforme (GBM) is the most common and aggressive form (CNS World Health Organization (WHO) grade 4), and it is characterized by a high proliferation rate and diffuse infiltration into surrounding brain tissue, with a poor prognosis and a median overall survival of approximately 20 months [1,2,3,4,5,6,7,8]. Current standard of care therapy includes total surgery resection, radiotherapy, and temozolomide (TMZ) chemotherapy. However, GBM is often resistant to treatment [2,3,4,8,9]. Due to this evidence, the detection of further novel molecular markers seems to be of fundamental importance for the diagnosis of GBM and for developing future targeted therapies [8,10].

It is well known that an alteration of the “glycan coat”, which normally covers the cell surface, has emerged as an established hallmark of cancer [11,12]. In particular, altered expression of glycans such as sialic acids (Sias) seems to have a crucial role in tumorigenesis [12]. Sias represent a family of large-size negatively charged 9-carbon 2-keto-3-deoxy sugars. They are primarily found as terminal components of glycans in the glycoconjugates and are linked to galactose or N-acetyl-galactosamine via α-2,3 or α-2,6 linkage or to other Sias via α-2,8 or rarely α-2,9 linkages, resulting in mono-, oligo- and polymeric form called polysialic acid (PolySia) [13,14]. The monomeric or oligomeric Sias are present in glycoproteins and glycolipids (gangliosides), whereas the polymeric form is mostly linked to the transmembrane glycoprotein Neural Cell Adhesion Molecule (NCAM) [15,16]. Because of these characteristics, a wide repertoire of biological functions has been proposed for the Sias, such as the maintenance of cell membrane stability and the modulation of intercellular and intermolecular phenomena in various tissues of animals, including humans, during embryonic and adult life, in physiological and pathological [14,15,16,17,18,19,20,21]. In such a context, several enzymes are involved in Sias synthesis (synthases), transfer on carbohydrate chain (sialyltransferases), and degradation (sialidases/neuraminidases) to maintain the normal and functional sialylation status in different types of tissues [22,23,24,25]. Several investigations have reported aberrant expression of monomeric and oligomeric Sias in various human cancer types, suggesting that they may represent potential biomarkers to distinguish cancer cells from their healthy counterparts [13,26,27,28,29,30,31,32,33,34,35,36]. Regarding PolySia, it is mainly expressed in developing tissues in physiological conditions, whereas it is almost absent in adult organs. Interestingly, PolySia is re-expressed or over-expressed in several cancerous cells [12,13,15,16,19,21,25,26,28,30,31,32,36,37]. Notably, it has been shown that an altered sialylation of cancer cells promotes cancerous growth, invasiveness, metastasis, immune evasion and drug resistance, meaning strategies to block aberrant sialylation on cancers will be highly beneficial [8,12,25,36,38,39].

To our knowledge, limited investigations on sialylation status and role in GBM have been performed and data results are sometimes discording, especially regarding the expression of monoSias and their correlated sialyltransferases [1,2,3,7,8,11,25,40]. In fact, some studies demonstrated that α-2,3-sialylated glycans are more highly expressed in GBM with respect to α-2,6-sialylated glycans [2,25]. In addition, α-2,3 sialylation has been found to promote progression, while α-2,6 sialylation limits GBM aggressiveness [11,40]. Instead, in another study, GBM cells with high α2,6 sialylation exhibited increased in vitro growth and self-renewal capacity and decreased mouse survival when orthotopically injected [7]. Therefore, a potential role of α-2,6 sialylation in gliomagenesis has been hypothesized [7]. A few literature data have shown high levels of PolySia in human GBM models, which seem to be associated with a less differentiated state of cells and a greater motility as well as an increase of tumor spreading in the brain parenchyma [1,3]. Noteworthy, an investigation on several different cell lines revealed different gene expression of sialyltransferases among cells, then resulting in different sialylation status, highlighting the complexity of the system [8].

Given the importance of the altered sialylation status in the progression of cancer cells, specific inhibitors have been developed to selectively target Sia biosynthesis. Among others, 3-Fax-peracetyl-Neu5Ac (3-Fax) has shown promising potential in its capability to efficiently block sialylation in many cancer types [3,25]. In fact, 3-Fax administration results in production of CMP-3-Fax-Neu5Ac, which is a competitive inhibitor of virtually all sialyltransferases and also causes negative feedback inhibition for the synthesis of CMP-Neu5Ac, thus resulting in the global blockade of sialylation [41,42,43,44]. 3-Fax has been shown to affect GBM cell vitality, migration and differentiation in specific circumstances [3,25].

On these premises, we decided to analyze the expression levels of sialyltransferases in silico and in vitro GBM models. Then we investigated the role of the sialylation status on GBM tumor properties, such as proliferation, migration and adhesion.

## 2. Results

### 2.1. Sialyltransferases Up-Regulation Correlates with Poor Survival in GBM Patients

A few literature data exist on the characterization and role of sialylation in GBM models. In order to perform a large scale analysis of sialyltransferases expression in human GBM tissues we took advantage of the R2 Genomics Analysis and Visualization Platform [45]. In detail, we analyzed non-tumoral (*n* = 36) and GBM (*n* = 386) samples from two GBM public datasets, deposited at http://r2.amc.nl (accessed on 2 June 2024) querying for the expression of sialyltransferases transcripts [46,47].

**Figure 1:**
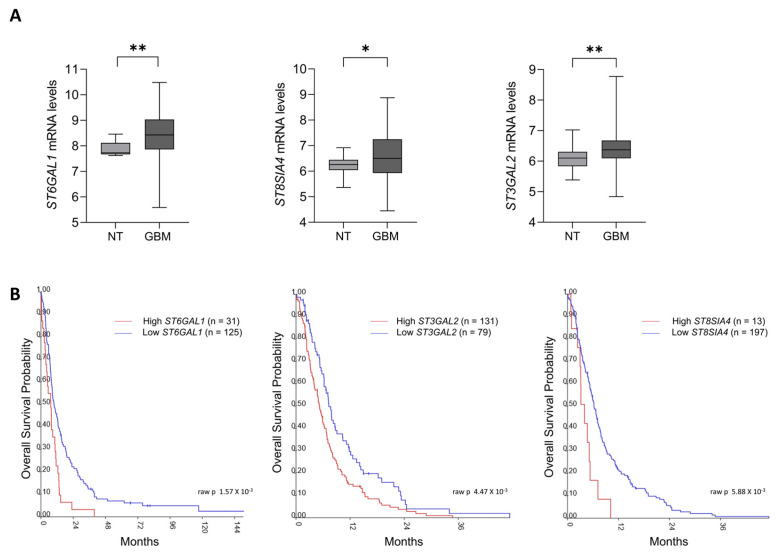
Increase in *ST6GAL1*, *ST3GAL2* and *ST8SIA4* mRNAs correlates with poor survival in GBM patients. (**A**) mRNA expression of *ST6GAL1*, *ST3GAL2* and *ST8SIA4* in non-tumoral (NT, *n* = 36) and GBM (*n* = 386) derived from both publicly available dataset French (276 total samples from Erasmus University Medical Center, Affymetrix HU133 Plus 2.0 arrays), and REMBRANDT–Madhavan (550 total sample from Georgetown Database of Cancer (G-DOC)). Data were analyzed by un unpaired-t test (* *p* < 0.05; ** *p* < 0.01). (**B**) Kaplan–Meier analysis for cohort of patients according to *ST6GAL1*, *ST3GAL2* and *ST8SIA4* expression levels in French and REMBRANDT dataset. Low *ST6GAL1 n* = 125; High *ST6GA1 n* = 31. *p* = 1.57 × 10^3^; expression cut-off 550.9. Low *ST3GAL2 n* = 79; High *ST3GAL2 n* = 131. *p* = 4.47 × 10^3^; expression cut-off 78.40 Low *ST8SIA4 n* = 197; High *ST8SIA4 n* = 13. *p* = 5.88 × 10^3^; expression cut-off 233.5.

Our analysis showed that the transcript levels of Golgi sialyltransferases β-galactoside α-2,6-sialyltransferase 1 (*ST6GAL1*), β-galactoside α-2,3-sialyltransferase 2 (*ST3GAL2*), and the polysialic acid-producing enzyme (*ST8SIA4*) are significantly up-regulated in the tissues of GBM patients compared to non-tumoral ones (Figure 1A). We also correlated mRNA levels of each sialyltransferase with patient overall survival. Intriguingly, Kaplan Meier analysis revealed poorer survival for patients with higher levels of either *ST6GAL1*, *ST3GAL2* and *ST8SIA4* (Figure 1B), similarly to what observed in other cancer models [12].

### 2.2. GBM Cell Lines Express Differential Levels of Sialyltransferases and Sias

**Figure 2:**
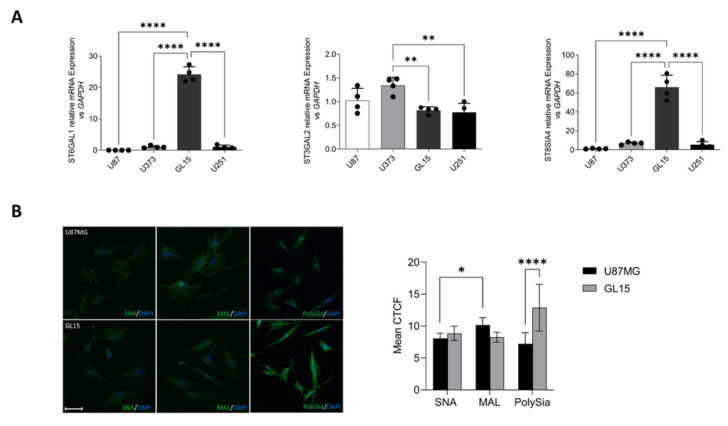
Characterization of the sialylation status in some GBM cell lines. (**A**) Real-Time PCR analysis showing mRNA expression of *ST6GAL1*, *ST3GAL2* and *ST8SIA4* in different GBM cell lines. *GAPDH* was used as internal control. Dots indicate the single values obtained. Statistical significance: ** *p* < 0.01; **** *p* < 0.0001, one-way ANOVA. (**B**) U87MG and GL15 were subjected to lectin cytofluorescence staining (green) to detect Sias linked to galactose or N-acetyl-D-galactosamine via α-2-6 linkage (SNA) and Sias linked to galactose via α-2-3 linkage (MAL I) and were also subjected to immunocytochemistry for PolySia. DAPI was used to stain nuclei (blue). Representative images of at least 10 fields showing the merged signals are shown. Scale bar: 20 μm. Analysis of the mean corrected total cell fluorescence (CTCF) was performed by ImageJ software (https://imagej.net/ij/, accessed on 2 June 2024) on 10 fields per each condition, and the corresponding graph is shown. Statistical significance: * *p* < 0.05; **** *p* < 0.0001, two-way ANOVA.

Prompted by the in silico analyses, we decided to investigate the in vitro expression of ST6GAL1, ST3GAL2 and ST8SIA4 in GBM cell models, namely U87MG, U373, GL15 and U251 cell lines. qPCR showed high variability of mRNA expression of the selected enzymes among the tested cell lines, with GL15 cells expressing the highest amount of both *ST6GAL1* and *ST8SIA4*, whereas U87MG the lower levels of the same sialyltransferases (Figure 2A). Therefore, we decided to characterize the different types of Sias expressed on the cell surface of GBM cells expressing the highest and lowest levels of the enzymes, namely GL15 and U87MG. Cytochemistry was performed using specific lectins that recognize Sias linked to galactose via α2-3 linkage (MAL I, *Maackia amurensis* lectin I), or that link to galactose or N-acetyl-D-galactosamine via α2-6 linkage (SNA, *Sambucus nigra* lectin). Lectin fluorescence staining revealed a higher α2-3-linked Sias content than α2-6-linked Sias on cell surface of U87MG cells under basal conditions, in line with data in the literature on other GBM in vitro models [25] whereas GL15 showed similar levels for both Sias (Figure 2B). We also analyzed the expression of PolySia in our models by using a specific anti-PolySia antibody. Consistently with qPCR, GL15 cells expressed a significantly higher PolySia content than U87MG cells (Figure 2B).

### 2.3. Sialylation Inhibition Doesn’t Affect GBM Cell Proliferation and TMZ Sensitivity

**Figure 3:**
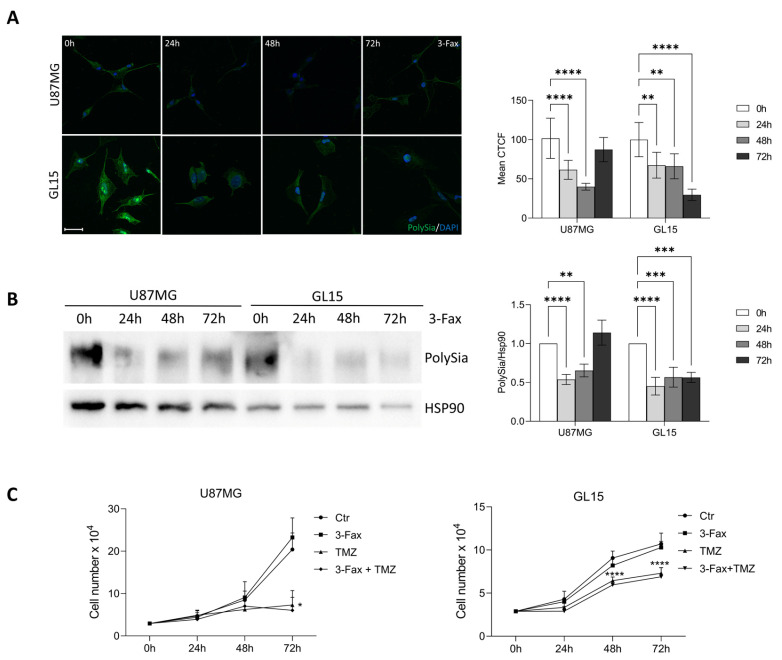
Suppression of the sialylation pathway doesn’t affect GBM cells proliferation and TMZ chemosensitivity. (**A**) Immunofluorescence analysis showing the time course (0–72 h) of sialylation pathway inhibition by 3-Fax in U87MG and GL15 cell lines. PolySia expression is visualized in green. DAPI was used to stain nuclei (blue). Scale bar: 20 μm. The graph represents the mean corrected total cell fluorescence (CTCF). Statistical significance: ** *p* < 0.01; **** *p* < 0.0001, two-way ANOVA. ImageJ software was employed to analyze 10 fields per each condition. (**B**) Western blotting analysis of PolySia expression was performed in protein extracts from U87MG and GL15 cells cultured in 3-Fax presence for 24, 48 and 72 h. HSP90 was used as loading control. A representative image of three different experiments is shown. The corresponding densitometric analysis is shown. Statistical significance: ** *p* < 0.01, *** *p* < 0.001, **** *p* < 0.0001, Two-way ANOVA. (**C**) U87MG and GL15 cells were cultured in complete DMEM or in presence of 100 µm of 3-Fax, TMZ 10 µM, or both. At the indicated time point, the cells were trypsinized and counted in a Thoma chamber. The graph represents the means ± SEMs of three different experiments. Data were analyzed by two-way analysis of variance (ANOVA) followed by Tukey post hoc. Statistical significance: * *p* < 0.05; **** *p* < 0.0001.

It has recently been shown that GBM cells with a high content of Sias present a sustained cellular proliferation and a more aggressive phenotype than GBM cells with low Sias level [7]. By taking advantage of the pan-sialyltransferase inhibitor 3-Fax, we investigated the role of sialylation on cell proliferation of both U87MG and GL15. Firstly, we confirmed the inhibitory effect of 3-Fax on PolySia expression at different time points in both U87MG and GL15 cells, by immunocytochemical and western blotting analyses. As shown in Figure 3A,B, PolySia levels were strongly decreased in both cell lines upon 3-Fax treatment for 24, 48 and 72 h. Notably, the 3-Fax inhibitory effect is maintained up to 72 h in GL15 cells, but not in U87MG in which a partial recovery of PolySia expression was observed at 72h.

We then analyzed cell proliferation through cell counting experiments and observed that both parameters were not affected by exposure to 100 µM of 3-Fax up to 72 h in both U87MG and GL15 (Figure 3B). We also investigated possible effects of 3-Fax on sensitivity of cells to TMZ, the first line chemotherapeutic agent for GBM treatment, known to induce a G2-arrest in cells [48]. As shown in Figure 3C, we found that the combination of 3-Fax and TMZ did not result in an additive inhibitory effect on cell proliferation in both cell types. Overall, these results suggest that inhibiting Poly-Sialylation by 3-Fax does not affect cell proliferation nor sensitize cells to TMZ.

### 2.4. Sialylation Inhibition Affects Migration and Invasion Ability of GBM Cells

**Figure 4:**
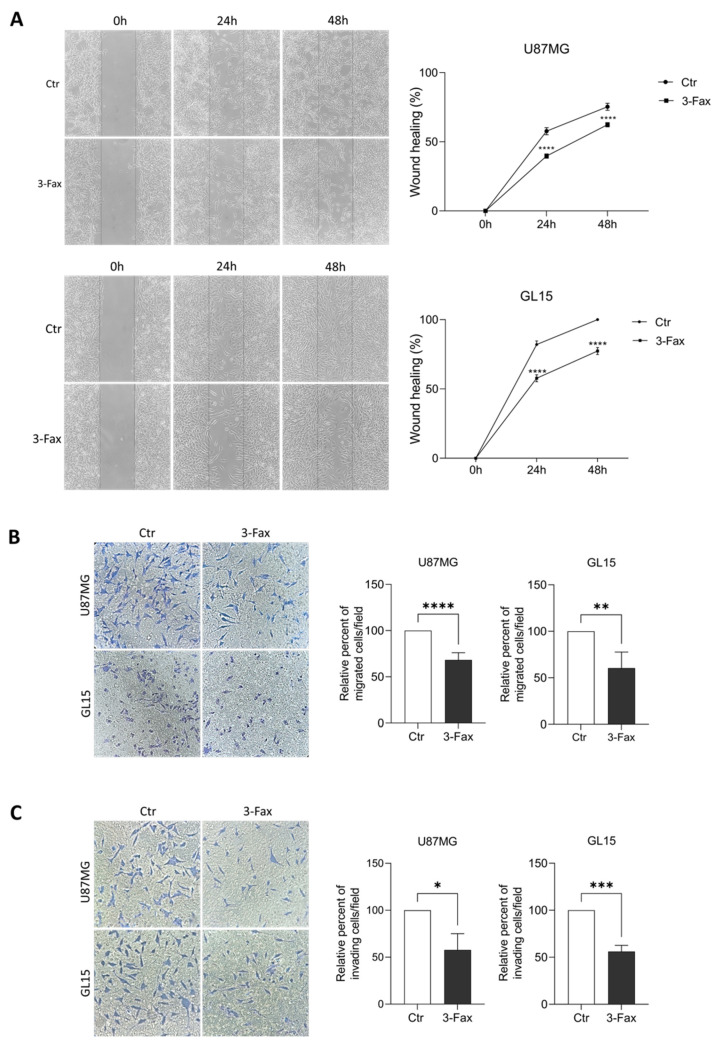
3-Fax impairs motility and migration ability of GBM cells. (**A**) Wound healing assay of U87MG and GL15 cell lines, grown in DMEM (Ctr), or in 3-Fax-containing medium (3-Fax). Phase-contrast pictures at 20× magnification at 0, 24 and 48 h after scratching and representative images of three independent experiments are shown. The wound healing area was analyzed by using ImageJ software and the corresponding data, relative to 0 h, are expressed in the graph. Statistical significance: **** *p* < 0.0001, two-way ANOVA. (**B**) Transwell migration assay was performed on U87MG and GL15 cells grown in DMEM (Ctr) or in 3-Fax-containing DMEM for 2 h. FBS was employed as a chemoattractant. The graph represents the mean ± SEM of the relative number of migrated cells counted in three different experiments. Statistical significance: ** *p* < 0.01; **** *p* < 0.0001 according to Unpaired *t*-test. (**C**) Invasion assay was performed on U87MG and GL15 cells grown in DMEM (Ctr) or in 3-Fax-containing DMEM. FBS was employed as a chemoattractant. The graph represents the mean ± SEM of the relative number of invading cells counted in three different experiments. Statistical significance: * *p* < 0.05; *** *p* < 0.001 according to Unpaired *t*-test.

Since it has been reported that the degree of sialylation of tumor cells correlates with their mobility and capacity to metastasize, we studied the effects of inhibition of the sialylation pathway on migration ability of U87MG and GL15 cells. At first, we performed a wound healing assay on both cell lines and observed a significant slowing down of mobility when cells were pre-incubated with 3-Fax for 48 h, in comparison to untreated cells (Figure 4A). We also tested the chemotactic capability of cells in presence of the sialylation inhibitor in transwell migration experiments. In line with the wound healing results, 3-Fax pre-incubation induced a significant reduction in the number of migrated cells, in response to a chemotactic gradient, compared to untreated cells (Figure 4B). In order to investigate the effect of 3-Fax on cell invasiveness, we performed transwell assays in presence of Matrigel and observed a significant reduction of cell invasion in 3-Fax cultured cells (Figure 4C).

Overall, these results show that the inhibition of sialylation by 3-Fax significantly impairs the migration and invasion ability of GBM cells.

### 2.5. Sialylation Inhibition Enhances Cell-Matrix Adhesion in GBM Cells

**Figure 5:**
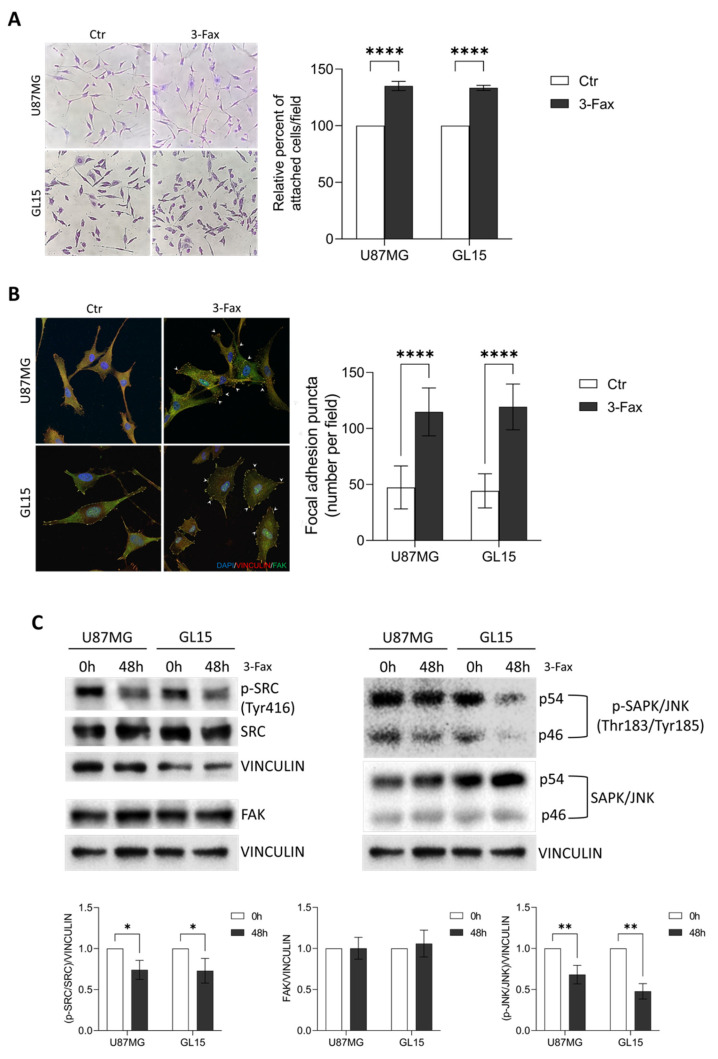
The inhibition of the sialylation pathway results in enhanced cell-matrix and cell-cell adhesion. (**A**) U87MGs and GL15 were treated with 100 µM of 3-Fax for 48 h or untreated (Ctr), then were fixed and stained by Crystal violet staining solution after trypsinization after 5 h-attachment to cell plate. The graph represents the mean ± SEM of the relative number of attached cells counted in three different experiments. Statistical significance: **** *p* < 0.0001 according to two-way analysis of variance (ANOVA) followed by Tukey post hoc. (**B**) Immunocytochemistry and confocal analysis for Vinculin (red) and FAK (green) was performed on U87MG and GL15 cells. DAPI was used to stain nuclei (blue). Arrowheads indicate Vinculin/FAK positive focal adhesion. Scale bar: 10 µm. The graph represents the mean ± SEM of the number of focal adhesion puncta (FAK/Vinculin double positive structures) counted per each field in three different experiments. Statistical significance: **** *p* < 0.0001 according to two-way analysis of variance (ANOVA) followed by Tukey post hoc. (**C**) Western blotting analysis of FAK, phospho Tyr416-SRC (p-SRC) and SRC expression was performed in protein extracts from U87MG and GL15 cells cultured in 3-Fax presence for 48 h (48h) or untreated (0 h) (**left panels**). Western blotting of phopsho Thr184/Tyr185 SAPK/JNK (p-SAPK/JNK) and total SAPK/JNK was performed in cells cultured in 3-Fax presence for 48 h (48h) or untreated (0 h) (**right panels**). Vinculin was used as loading control. Representative images of three different experiments are shown and the corresponding densitometric analyses are shown. Statistical significance: * *p* < 0.05, ** *p* < 0.01, Multiple Unpaired *t*-test.

Given the anti-migratory effects of 3-Fax observed on our GBM cell models, we then performed an adhesion assay to test whether the decrease in mobility was coupled to an increased adhesiveness. As shown in Figure 5A, both U87MG and GL15, incubated with 3-Fax for 48 h, showed significantly greater ability to re-attach to the cell plate after trypsinization compared to control cells.

Both cell adhesion and migration are tightly ruled by several players, such as focal adhesion proteins, i.e., Vinculin and focal adhesion protein (FAK), molecular motor proteins and cytoskeleton. Thus, we decided to investigate the effects of 3-Fax on the ability of cells to form focal adhesions. Co-immunostaining experiments showed a significant increase of Vinculin/FAK-positive structures, namely focal adhesions, at the plasma membrane for both cell lines when compared to untreated cells (Figure 5B).

To better investigate the effect of desialylation on cell adhesion, we explore FAK signaling by analyzing the phosphorylation level of two FAK-related protein kinases, SRC and SAPK/JNK [49]. Activation of FAK by integrin clustering, indeed, leads to autophosphorylation and SRC binding. Once activated, FAK and SRC form a functional complex initiating a signaling cascade which is crucial for the dynamic regulation of focal adhesions and that also culminates with activation of several other protein kinases including JNK. Both SRC and JNK activation are compromised in 3-Fax cultured cells, thus demonstrating an impairment of FAK signalosome (Figure 5C), as already observed [50].

These results suggest that sialylation is a crucial mechanism in regulating cell migration and adhesion in GBM cells.

### 2.6. Sialylation Inhibition Affects Migration and Invasion Properties of Primary GBM Cells

**Figure 6:**
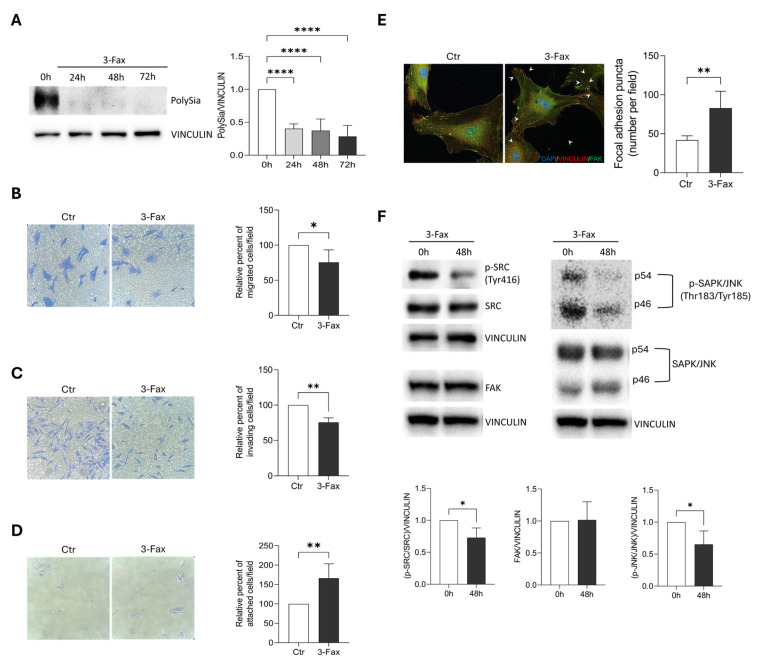
Analysis of migration and invasion in a GBM primary cell line upon sialylation inhibition by 3-Fax. (**A**) Western blotting analysis of PolySia expression was performed in protein extracts from GL18-15 cells cultured in 3-Fax presence for 24, 48 and 72 h. VINCULIN was used as loading control. A representative image of three different experiments is shown. The corresponding densitometric analysis is shown in the graph. Statistical significance: ****p < 0.0001, One-way ANOVA. (**B**) Transwell migration assay was performed on GL18-15 cells grown in DMEM (Ctr) or in 3-Fax-containing DMEM for 2 h. FBS was employed as a chemoattractant. The graph represents the mean ± SEM of the relative number of migrated cells counted in three different experiments. Statistical significance: * *p* < 0.05 according to Unpaired *t*-test. (**C**) Invasion assay was performed on GL18-15 cells grown and stimulated as in (**B**). The graph represents the mean ± SEM of the relative number of invading cells counted in three different experiments. Statistical significance: ** *p* < 0.01 according to Unpaired *t*-test. (**D**) GL18-15 cells, treated as in (**B**), were plated and fixed after 5 h, then stained with Crystal violet solution. The graph represents the mean ± SEM of the relative number of attached cells counted in three different experiments. Statistical significance: ** *p* < 0.01 according to Unpaired *t*-test. (**E**) Immunocytochemistry and confocal analysis for Vinculin (red) and FAK (green) was performed on GL18-15 cells treated as in (**B**). DAPI was used to stain nuclei (blue). Arrowheads indicate FAK/Vinculin double positive structures (focal adhesions). Scale bar: 10 µm. The graph represents the mean ± SEM of the number of focal adhesion puncta counted per each field in three different experiments. Statistical significance: ** *p* < 0.01 according to Unpaired *t*-test. (**F**) Western blotting analysis of FAK, phospho Tyr416-SRC (p-SRC) and total SRC, phopspho Thr184/Tyr185 SAPK/JNK (p-SAPK/JNK) and total SAPK/JNK was performed on GL18-15 cells cultured in 3-Fax presence for 48 h (48 h) or untreated (0h). Vinculin was used as loading control. Representative images of three different experiments are shown and the corresponding densitometric analyses are shown. Statistical significance: * *p* < 0.05 Unpaired *t*-test.

To confirm the results obtained on GL15 and U87MG cells, we extended our analysis to GBM primary cells (GL18-15) derived from a patient after surgical resection and already characterized for ST expression in previous studies [3]. Similarly to what observed in U87MG and GL15 cells, 3-Fax treatment strongly reduced PolySia in GL18-15 (Figure 6A). Migration and invasion capabilities were also reduced in GL18-15 pre-incubated with the inhibitor for 2 h (Figure 6B,C). As seen for U87MG and GL15, GL18-15, incubated with 3-Fax for 48 h, showed significantly greater ability to re-attach to the cell plate after trypsinization compared to control cells (Figure 6D). We also explored the presence of FAK/Vinculin positive sructures by immunofluorescence analysis and found a significant increase in 3-Fax-treated cells in comparison to untreated ones (Figure 6E). Similarly to U87MG and GL15, FAK signaling appeared compromised, as demonstrated by a reduced phosphorylation of SRC and SAPK/JNK kinases (Figure 6F). Overall, we were able to reproduce, in GBM primary cells, the results obtained in cell lines upon pharmacological inhibition of sialylation.

## 3. Discussion

In recent years, an altered “glycan coat” normally covering the cell surface has emerged as an established hallmark of cancer [11,12]. Notably, a marked altered expression of the sialic acids (Sias) has been associated with many types of high-grade tumors, characterized by aggressiveness and an undifferentiated state [12]. Even in the context of glioblastoma multiforme (GBM), the most common primary and lethal brain tumor in humans, glycobiology has recently attracted considerable interest and is emerging as a promising area of biological research and a potential therapeutic target [51]. However, regarding the sialylation status and its role in GBM, data are still limited and sometimes discordant [1,2,3,7,8,11,25,40]. Therefore, in this study we performed a well-targeted characterization of the sialylation status in GBM by evaluating specific sialyltransferases and various types of Sias in different GBM cell lines. In addition, using the pan-sialyltransferase inhibitor 3-Fax, we highlighted the role of sialylation in some of the main oncogenic features of GBM.

Firstly, querying the R2 genomic platform to investigate the mRNA expression level of the various sialyltransferases in GBM, we have elicited our attention on β-galactoside α-2,6-sialyltransferase 1 (*ST6GAL1*), β-galactoside α-2,3-sialyltransferase 2 (*ST3GAL2*), and the PolySia-producing enzyme *ST8SIA4*. Through in silico analysis we showed that mRNAs of only *ST6GAL1*, *ST3GAL2* and *ST8SIA4* are significantly upregulated in GBM tissues compared to non-tumor ones. We also showed that increased mRNA expression level of these sialyltransferases are related to a poorer patient survival rate. To our knowledge, only a few investigations have been previously performed and are partially in agreement with our data. In fact, Chong et al. showed that higher mRNA levels of *ST3GAL1* are associated with increased tumor grades of gliomas and lower patient survival [52]. On the other hand, in order to investigate the relevance of different α-2,3-sialyltransferases (*ST3GAL*s) in GBM, Putthisen et al. used in silico analyses to show an increased mRNA expression levels for *ST3GAL2*, *ST3GAL4*, *ST3GAL6*, and a slightly decrease for *ST3GAL1*, *ST3GAL3*, and *ST3GAL5* in GBM tissues, compared to non-cancer tissues [2]. However, Kaplan-Meier analysis showed no significant correlation between increased expression of *ST3GAL3* and *ST3GAL4* in GBM samples and shorter survival rate of patients [2].

Based on these findings, we conducted an analysis of the expression levels of the sialyltranserases of our interest in four different GBM cell lines and observed an high variability, as recently also observed by Scibetta et al. [53]. In detail, we highlighted that GL15 cells express the highest amount of both *ST6GAL1* and *ST8SIA4*, when compared to other GBM cell lines. Instead, the U87MG cells were found to express the lowest levels for both *ST6GAL1* and *ST8SIA4*. Since the overexpression of sialyltransferases leads to hypersialylation, we next conducted a characterization of the Sias expressed on the cell surface of GBM cells, focusing our attention to GL15 and U87MG cells due to the evident difference in *ST6GAL1* and *ST8SIA4* expression observed. Consistent with the mRNA expression levels of sialylation enzymes, GL15 cells were found to express significantly more PolySia than U87MG ones. α-2,6-linked Sias were also slightly increased in GL15 compared to U87MG, although not significantly. Of note, regarding α-2,3-linked Sias and *ST3GAL2*, a similar expression trend was detected in the two cell lines.

It is well established in the scientific community that a dysregulation of sialylation status in malignancies is mainly associated with tumor growth, metastasis and invasion [11,12,28,32]. Thus, we explored the effects of the inhibition of the sialylation pathway on GL15 and U87MG cells, to address the role of Sias in GBM tumor properties in two models expressing significantly different sialyltrasnferases levels. We exploited global sialyltransferase inhibition on both GL15 and U87MG cell lines using the pan-sialyltransferase inhibitor 3-Fax, delivered as a per-acetylated methyl ester pro-drug [44]. The mechanism by which 3-FAX inhibits sialyltrasnferases activity consists in mimicking and competing with their substrate; however, RNA analyses could be performed to exclude a potential effect on sialyltransferases gene expression.

After verifying the actual ability of 3-Fax to suppress sialylation pathway, by checking expression levels of PolySia, we performed some functional tests on the main oncogenic properties of GBM, such as proliferation, migration and adhesion. Despite the different degree of sialylation in the two cell lines, we obtained similar findings. In fact, 3-Fax was not able to influence the growth of both GBM cell lines either alone or in combination with TMZ, the first line drug for chemotherapeutic treatment, as already reported in a previous study [3] and as also observed in a different cancer model, such as melanoma [54]. However, we observed a significant reduction in mobility and migration capacity in both cell lines, associated with an increased ability of these cells to adhere to the substrate. In this regard, it has been also shown that sialylation pathway inhibition by 3-Fax in melanoma abrogates cell migration ability by impairing binding to poly-L-lysine, type I collagen, and fibronectin [54]. In line with this result, 3-Fax significantly reduced interactions of myeloma cells with E-selectin, MADCAM1 and VCAM1, suggesting that reduced sialylation impairs extravasation and retention of myeloma cells in the bone marrow, altering the post-translational modification of the α4 integrin [55]. Interestingly, some investigations showed that hyper-sialylation of some membrane receptors, due to ST6GAL1 overexpression, correlated with the ability of tumor cells to migrate and metastasize. β1 integrin [56,57], Fas and TNFR1 death receptors [58,59] and the receptor tyrosine kinases (RTK) EGFR, MET and HER2 [60,61,62,63,64] are ST6GAL1 targets. It is noteworthy that RTK receptors play a pivotal role in various cancer types, including GMB [65,66,67,68]. To exclude possible 3-FAX-induced unspecific effects, and to investigate the contribution of the single sialyltransferases, their genetic depletion could be also designed in future experiments.

In addition, it is well known that the polysialylated form of NCAM is associated to cancer progression. In fact, due to its anti-adhesive and repulsive characteristics as well as to its ability to modulate signaling, PolySia plays a determinant role in cancer cell detachment and metastasis [3]. The PolySia-mediated masking mechanism might also help neoplastic cells in eluding the immune system through the binding to specific immunoglobulin type lectins such as Siglecs. Infact, Siglecs are known to respond to specific glycan signatures by triggering tolerogenic or immunogenic signaling pathways, and then can favor cancer cell proliferation and metastasis [34,36].

Another interesting finding emerges from our investigation regarding increased adhesiveness of GBM cells upon 3-Fax treatment. In fact, we observed an increase in the formation of focal adhesion complexes. In particular, after treatment with 3-Fax we observed a significant increase in Vinculin-FAK-positive structures, also referred to as focal adhesion puncta, compared to the control condition. The focal adhesion complex is an integrin-containing multi transmembrane protein assembly composed of integrin, vinculin, tensin, talin, focal adhesion kinase, and paxillin that connects the intracellular cytoskeleton to the extracellular matrix and regulates cell adhesion, migration, diffusion, and differentiation [69]. Focal adhesion kinase (FAK) is considered as a hub in the interactome of focal adhesions complex [70,71,72]. Once activated, FAK helps to disassemble focal adhesions, the loss of which is necessary for the migration of normal and tumor cells. Recent evidence supports our finding of increased focal adhesion puncta following sialylation inhibition. Indeed, Devi et al. (2021) show a co-localization of GNE (UDP N-acetylneuraminic 2-epimerase/N-acetylmannosamine kinase), a key enzyme for Sia biosynthesis, with paxillin, associated with a reduced migratory capacity of cells overexpressing mutated GNE [73]. Overall, this evidence clearly suggests a role for Sias in driving molecular dynamics at focal adhesion sites that lead their assembly and disassembly in order to rule cellular mobility dynamics. Future experiments evaluating the impact of pharmacological and genetic desialylation on 3D GBM models such as spheroids and tumoroids will be crucial to confirm the in vitro results obtained in 2D models.

## 4. Materials and Methods

### 4.1. Analysis of Publicly Available Patients’s Transcriptomic Data

The R2 Genomics Platform (http://r2.amc.nl, accessed on 2 June 2024) was used to analyze transcriptomic datasets from non-cancer and Glioblastoma patients. We selected the following datasets: Tumor Glioma_French_284_MAS5.0_u133p2 (276 samples from Erasmus University Medical Center, analyzed using Affymetrix HU133 Plus 2.0 arrays), and Tumor Brain (REMBRANDT study)-Madhavan-550-MAS5.0-u133p2 (550 samples from the Georgetown Database of Cancer (G-DOC). For both datasets, we downloaded log2-transformed and normalized expression data using the Data Grabber function, selecting only non-tumoral (NT) and Glioblastoma (GBM) subgroups. We focused on the expression of *ST6GAL1*, *ST3GAL2*, and *ST8SIA4* transcripts. Graphs generations and statistical analysis were performed by using GraphPad Prism software (version 9, GraphPad, La Jolla, CA, USA). Survival analysis was carried out using the Kaplan Scan tool available online (https://hgserver1.amc.nl/cgi-bin/r2/main.cgi?option=kaplan_main, accessed on 2 June 2024). Patients were stratified into high and low expression groups based on calculated cut-off values for *ST6GAL1*, *ST3GAL2*, and *ST8SIA4*. Right-censored overall survival, at 5 or 12 years, was estimated using the Kaplan–Meier method, and significance was evaluated with the Gehan–Breslow–Wilcoxon test.

### 4.2. RNA Isolation and Quantitative RT-PCR

Total RNA was extracted using RNeasy kit (Qiagen, Hilden, Germany) according to the manufacturer’s instructions. One microgram of total RNA was reverse-transcribed using Superscript III reverse transcriptase (Thermo Fisher Scientific, Waltham, MA, USA, A11008) and the resulting cDNAs were amplified using Power SYBR Green PCR Master Mix (BioRAD, Hercules, CA, USA) following the manufacturers’ instructions. Quantitative PCR analysis was performed on a CFX Connect (BioRAD) as previously described by Pepe et al., (2023) [74]. The following primers were used (5′–>3′): ST3GAL2 Fw: GTGCCTCCGACTGGTTTG; ST3GAL2 Rv: GAAGCGGTAGGGGTTCTC; ST6GAL1 Fw: CTGAATGGGAGGGTTATCTGCC; ST6GAL1 Rv: ACCTCAGGACTGCGTCATGATC; ST8SIA4 Fw: CAAGAACTGAGGAGCACC; ST8SIA4 Rv: TTTCCAACCTTCTACATTGTG. Gene expression was normalized on GAPDH by using the following primers: GAPDH Fw: CATGTTCGTCATGGGGTGAACCA; GAPDH Rv: AGTGATGGCATGGACTGTGGTCAT.

### 4.3. Cell Culture and Treatments

The human GBM cell line U87MG was kindly provided by Prof. G. Velasco (Complutense University, Madrid, Spain), while the GL15 cell line was provided by Dr. E. Castigli (University of Perugia, Italy). The U373 cell line was provided by Prof. M. Segatto (University of Molise, Italy), while U251 cell line and GL18-15 patient-derived primary cells by Dr. P. Rosa (Sapienza University of Rome). U87MG, GL15, U373, U251, GL18-15 cell lines were cultured in high-glucose DMEM (Merck Life Science, Milan, Italy) and GL18-15 cells in DMEM/HAM’s F-12 (Merck Life Science, Milan, Italy), in presence of 10% heat-inactivated fetal bovine serum (FBS) (Merck Life Science, Milan, Italy) and 1% Penicillin/Streptomycin, and maintained at 37 °C in a humidified atmosphere containing 5% CO_2_. To inhibit sialyltransferases, 100 µM of 3-Fax-Peracetyl Neu5Ac (3-Fax) (566224, Merck Life Science, Milan, Italy) was added to the culture medium for 24, 48, and 72 h, as indicated. Cells treated with the vehicle, dimethyl sulfoxide (DMSO), served as controls. Furthermore, for the proliferation assay, 10 μM of Temozolomide (TMZ) (500609, Merck Life Science, Milan, Italy) was applied for the indicated time points.

### 4.4. Lectin Cytochemistry

For lectin staining, U87MG and GL15 cells were seeded on glass coverslips at a density of 80 × 10^3^ cells per coverslip and cultured under basal conditions without treatment. Cells were fixed with 4% paraformaldehyde (PFA) in PBS, and subsequently blocked with 3% BSA in PBS for 1 h. Lectin cytochemistry was performed using *Maackia amurensis* lectin I (MAL I) (1:500, Vector Laboratories, FL-1311-2) to detect α2,3-linked Sias to galactose, and *Sambucus nigra* lectin (SNA) (1:500, Vector Laboratories, FL-1301) for α2,6-linked Sias to galactose or N-acetyl-D-galactosamine. Incubation with lectins was carried out for 1 h on ice. After DAPI (D9542, Merck Life Science, Milan, Italy) counterstaining, coverslips were mounted using Fluoroshield mounting medium (Invitrogen, Darmstadt, Germany, Cat. P36984). Images were captured using a Leica TCS SP8 confocal microscope (TCS SP8; Leica, Wetzlar, Germany) equipped with 63× objective and processed using Leica LAS X software (version 3.5.5) for Windows 10 (Leica Camera, Wetzlar, Germany).

### 4.5. Immunocytochemistry

U87MG and GL15 cells were seeded on coverslips at a density of 80 × 10^3^ cells per coverslip and grown in DMEM supplemented with 10% FBS. Following treatment, cells were fixed with 4% paraformaldehyde (PFA) in PBS, permeabilized with 0.1% Triton X-100 for 5 min, and blocked with 3% bovine serum albumin (BSA) in 0.1% PBS-Triton X-100 for 1 h. Cells were then incubated overnight at 4 °C with the following primary antibodies: anti-poly-Sia (1:100, Thermo Fisher Scientific, MA5-47855), anti-FAK (1:100, Santa Cruz Biotechnology, Dallas, TX, USA, SC-932), and anti-Vinculin (1:50, Santa Cruz Biotechnology, SC-73614). After washing, samples were incubated for 1 h at room temperature with goat anti-rabbit secondary antibody Alexa Fluor 488 (Thermo Fisher Scientific, Waltham, MA, USA, A11008) and goat anti-mouse secondary antibody Alexa Fluor 555 (Thermo Fisher Scientific, Waltham, MA, USA, A28180). Nuclei were counterstained with DAPI (D9542, Merck Life Science, Milan, Italy), and coverslips were mounted with Fluoroshield mounting medium (Thermo Fisher Scientific, Waltham, MA, USA, P36984). Images were acquired using a Leica TCS SP8 confocal microscope (Leica, Wetzlar, Germany) equipped with a 63× objective and processed using Leica LAS X software (version 3.5.5) for Windows 10 (Leica Camera, Wetzlar, Germany).

### 4.6. Proliferation Assay

U87MG and GL15 cells were seeded at a density of 30 × 10^3^ cells/well in 24-well plates and grown in DMEM supplemented with 10% FBS and then incubated at 37 °C in a 5% CO_2_ environment. After treatment with 10 µM TMZ, 100 µM 3-Fax, their combination, or DMSO vehicle control, as described above, cell proliferation was assessed by manual counting using a Blutzählkammer THOMA chamber (Merck Life Science, Milan, Italy) at 0, 24, 48, and 72 h after cell detachment with trypsin.

### 4.7. Wound Healing Assay

A wound-healing assay was used to compare the migratory ability of GL-15 and U87MG cells under control conditions and following treatment with 3-Fax. In particular, 50 × 10^3^ cells were seeded into the inner wells of cell culture inserts (Ibidi GmbH, Grafelfing, Germany) placed in Petri dishes. After cell attachment, the inserts were removed, creating a 500 µm cell-free gap to allow for cell migration. Cells were then cultured in low serum (0.5% FBS) medium, with or without 100 µM 3-Fax. To analyze cell migration, the wounded areas were photographed at the indicated time points with CoolSNAP camera (Photometrics) coupled to an ECLIPSE TieS phase contrast microscope (Nikon Microscopy, Melville, NY 11747-3064, USA) and processed using ImageJ analysis software (NIH). The percentage of wound healing was calculated as follows: [1 − (empty area X h/empty area 0 h)] × 100.

### 4.8. Transwell Migration and Invasion Assay

U87MG and GL15 cells were trypsinized and collected, then pre-incubated in suspension for 1h at 37 °C in invasion medium (DMEM without FBS, supplemented with 1% Penicillin/Streptomycin, 0.1% BSA and 25 mM HEPES, pH 7.4), in the presence or absence of 3-Fax. After pre-incubation, cells were seeded onto 12-well Transwell inserts (8 µm pore size, Sarstedt, Nümbrecht, Germany) at a density of approximately 15 × 10^3^ for U87MG, while 20 × 10^3^ for GL-15. In invasion assays, cells were seeded on transwell inserts coated with Matrigel (CLS354234, Merck Life Science, Milan, Italy), as previously reported [75]. A chemotactic gradient was established by adding DMEM supplemented with 10% FBS to the lower chamber, while the upper chamber (where cells were seeded) contain FBS-free invasion medium with or without 3-Fax. Following 24 h of incubation at 37 °C and 5% CO_2_ in a humidified atmosphere, the cells were fixed with ice-cold 10% trichloroacetic acid (TCA) for 10 min. Non-migrated cells remaining on the upper chamber were gently removed using a cell scraper. Migrated cells adhering to the underside of the insert membrane were stained with a solution containing 50% isopropanol, 1% formic acid, and 0.5% Brilliant Blue R-250 (Sigma-Aldrich, St. Louis, MO, USA). Images were acquired using a Nikon Eclipse 7s100 microscope (Nikon Europe, Amstelveen, The Netherlands) at 20× magnification. At least 10 random fields per insert were captured and manually counted to quantify cell migration and invasion.

### 4.9. Cell Adhesion Assay

U87MG and GL15 cells at least 60–70% confluence were trypsinized, and pre-incubated with vehicle or 3-Fax in invasion medium (DMEM supplemented with 1% Penicillin/Streptomycin, 0.1% BSA and 25 mM HEPES, pH 7.4) for 3 h and seeded on culture dishes with or without treatment. The cells were allowed to adhere to the cell plate surface for 5 and 18h. The non-adherent cells were removed with several washes in PBS, while the plate-adherent cells were fixed and stained with a solution containing 0.25% Crystal Violet in 20% methanol (Sigma-Aldrich, St. Louis, MO, USA) and counted under a microscope (Nikon Eclipse 7s100; Europe, Amstelveen, The Netherlands) on 10 fields at 20× magnification.

### 4.10. Western Blotting and Antibodies

Protein extracts were prepared by lysing cells with an appropriate amount of RIPA Buffer (50 mM Tris–HCl pH 8.0, 150 mM NaCl, 1% Nonidet P-40, 1 mM EDTA, 0.5% sodium deoxycholate, 0.1% SDS) supplemented with protease inhibitors cocktail. Samples were then incubated on ice for 20 min, samples were centrifuged at 160,000× *g* 15 min at 4 °C. Supernatants were recovered and protein concentrations were determined using Lowry protein assay (Bio-Rad Laboratories, Milan, Italy). Laemmli Buffer 4X (Thermo Scientific Chemicals, J60015.AD) was added to supernatants and the samples boiled at 70 °C for 10 min. Proteins were separated on SDS-PAGE gel and electroblotted onto nitrocellulose membranes (GE Healthcare, Life Sciences, Little Chalfont, Buckinghamshire, UK). Blot membranes were then incubated with primary antibodies in 5% nonfat dry milk in PBS/0,1% Tween-20 overnight at 4 °C. Detection was obtained by using horseradish peroxidase-conjugated secondary antibody (Bio-Rad Laboratories, Milan, Italy) and immunocomplexes were visualized with ECL plus (GE Healthcare, Life Sciences, Little Chalfont, Buckinghamshire, UK, WBULS0500) and chemiluminescence was recorded using ChemiDoc MP system (Bio-Rad Laboratories, Milan, Italy). The following primary antibodies were used: anti-poly-Sia (1:1000, MyBioSource, San Diego, CA, USA, MBS-488177) anti-Hsp90 (1:5000, Santa Cruz Biotechnology, SC-1346), anti-FAK (1:1000, Cell Signaling Technology, Danvers, MA, USA, CS-3285) anti-FAK (1:1000, Santa Cruz Biotechnology, SC-932), and anti-Vinculin (1:5000, Santa Cruz Biotechnology, SC-73614), anti-phopsho SRC (Tyr416, 1:1000, Cell Signaling Technology, CS-2101), anti-SRC (1:1000, Cell Signaling Technology, CS-2108), anti-phospho SAPK/JNK (Thr183/Tyr185, 1:1000, Cell Signaling Technology, CS-4671) and anti-SAPK/JNK (1:1000, Cell Signaling Technology, CS-9252).

### 4.11. Statistical Analysis

All the experiments were performed at least three times. Statistical analysis was performed using Graphpad Prism 9 software (GraphPad, La Jolla, CA, USA). All data were presented as means ± standard deviations (SDs). Statistical significance was determined, as indicated, using unpared-t test and one-way or two-way analysis of variance (ANOVA) followed by Tukey’s post hoc test. Differences are considered statistically significant (*) at *p* < 0.05.

## 5. Conclusions

Overall, our in silico analyses indicate a clear correlation of expression of specific sialyltransferases and patient survival rate. By means of in vitro experiments we showed that an aberrant expression of different Sias types is crucial for cell migration and adhesion ability of GBM cells. In addition, we highlighted for the first time an involvement of sialylation status in stabilize/destabilize FAK in focal adhesion complexes. Our findings suggest Sias and specific sialyltransferases as crucial molecular targets exploitable to design innovative therapeutic strategies for GBM. Further analyses, such, in example, the genetic knockdown of specific STs, will be necessary to investigate the molecular mechanisms and the specific molecules involved. Evaluating the impact of pharmacological desialylation on 3D GBM models such as tumoroids will be necessary to confirm the in vitro results obtained in 2D models. Lastly, potential off-target effects of pharmacological inhibitors of sialylation should be truly investigated, to exclude possible aspecific effects.

## Data Availability

The original contributions presented in this study are included in the article. Further inquiries can be directed to the corresponding authors.
